# Comparison of Surgery test scores using Concept Maps and Interactive Lectures among the Undergraduate Medical Students

**DOI:** 10.12669/pjms.40.10.10526

**Published:** 2024-11

**Authors:** Zubia Masood, Masood Jawaid, Syed Moyn Aly, Zubair Muhammad

**Affiliations:** 1 Zubia Masood, MBBS, FCPS (Surgery), MHPE. Associate Professor of Surgery, Baqai Medical University, Karachi Pakistan; 2 Masood Jawaid, MBBS, MCPS, FCPS (Surgery), MHPE Director Medical Affairs, PharmEvo; 3 Syed Moyn Aly, MBBS, MHPE, PhD Scholar Director Academics, Jinnah Sindh Medical University (JSMU), Karachi Pakistan; 4 Zubair Muhammad, MBBS, FCPS, FRCS, FACS, MHPE. Professor of Surgery, Dow University of Health Sciences, Karachi Pakistan

**Keywords:** Instructional strategy, Concept maps, Interactive lecture, Surgery

## Abstract

**Objective:**

Concept Maps (CM) aid in the acquisition of new information, and comprehend prior knowledge with better retention. Their use is not explored in undergraduate surgical education in our country, hence this study aimed to compare the surgery test scores of undergraduate medical students taught by concept maps and interactive lectures.

**Methods:**

This quasi-experimental study was carried out at the Department of Surgery, at a Private University Hospital among 60 students of fourth year MBBS from 2017-2018. The students were divided into Group-A taught by interactive lectures and Group-B taught by CM. Post class Multiple Choice Questions test was taken and scores were compared for immediate effects on knowledge and short term knowledge retention using the Unpaired t- test. A P-value of < 0.05 was considered significant.

**Results:**

Our study reported a mean test score of 19.5 (2.75 ± 0.49) for the Group-A, while 21.1 (3.49 ± 0.65) for Group-B with a negative t value of 2.003 with a significant p-value of 0.05. This showed that the test scores of Group-A were lower than the scores of Group-B.

**Conclusions:**

Concept Maps is an effective and promising way to give learners better understanding of complex concepts in short amounts of time. providing valuable evidence for instituting the use of concept maps as a continuous teaching strategy for medical students.

## INTRODUCTION

In this forever progressing medical environment, education has also advanced towards surpassing from traditional teacher-focused teaching to more student-centered methodologies which actively interact and engage students in the learning process creating a productive learning environment. Interactive lectures are found to augment interchange among the teachers, students and the lecture content endorsing active learning.^1^ Due to rapidly evolving scientific information, students encounter continuously accumulative knowledge during their courses making retention and recall a challenge. Proficiency to comprehend concepts and interconnect them to prior knowledge results in meaningful learning. Critical thinking, clinical reasoning, and problem solving are essential skills to be obtained by medical students during their education for becoming competent, proficient, and knowledgeable future practitioners.^1^,^2^

Above mentioned issues calls for the need of novel teaching strategies, enabling teachers to cover important curricular contents within the given time and medical students to learn new concept concepts with and recalling previous knowledge. Concept maps (CM) is one such teaching tool developed by Novak^3^ and adapted to medical education by Daley and Torre^4^ using graphics for organizing and representing knowledge. Based on Ausubel theory of meaningful learning,^3^ they are said to assist learners in assimilating lengthy theoretical information into brief and useable information in the form of pictographs based on their prior knowledge.^3^-^5^

CM is a graphical demonstration of a concept and the complex relationships among different concepts hierarchically. Concepts are in closed in nodes, classically illustrated by words enclosed in geometrical shapes like squares, ovals, circles or rectangles.^3^ The networks among the concepts are denoted by a linkage or line with an arrow to join numerous concepts. With this tool learning new concepts is linked to the learner’s prior concepts and knowledge, enabling the learner to intentionally link, differentiate and then correlate one concept to another.^3^,^4^ It not only offers the opportunity for the students to breakdown the novel information into smaller chunks, assemble these concepts to make logic but also helps in making useful associations between the concepts.^4^,^5^

This practice marks in significant long-lasting and easy-to-retain learning. Apart from these CM provides several opportunities to students for making relevant and key connections within the body of knowledge, clarifies misconceptions, enhances interest, encourages the generation of new ideas, promotes critical thinking, creativity, problem-solving and integrating theory to practice.^2^-^5^ CM facilitates student collaboration and fosters meaningful learning rather than rote learning.^6^ CM have been used effectively in schools children and science students for clarification and learning of difficult concepts.^7^-^9^

In nursing education CM are well explored. Their utilization have been found to improve critical thinking, clinical reasoning, problem-solving, patient-centered holistic care, academic performance, overall competence and student satisfaction.^10^-^13^ Limited work has been published in the medical and dental basic sciences education while there is gap in knowledge in clinical subjects particularly in the field of surgery both at national and international level.^14^-^17^ To the best of our knowledge no such work was published in general surgery from Pakistan.^14^-^17^ Keeping in view the above findings, our study aimed to compare the surgery test scores of undergraduate medical students taught by concepts concept maps and interactive lectures.

### Operational Definitions:

### Concept Maps (CM):

A concept map is a graphic representation of a concept and the compound connections among different concepts. Concepts are encircled in nodes, typically illustrated by words encircled in geometric shapes like squares, circles, rectangles or ovals. The connections among the concepts are symbolized by a line or linkage with an arrow to associate numerous concepts.^3^

### Meaningful learning:

It is the capability of understanding and correlating appropriate medical concepts by connecting them to the knowledge gained previously.^3^

## METHODS

This quasi-experimental study was conducted among Fourth year MBBS students at Department of Surgery, Ziauddin University Hospital from 2017- 2018. Sixty students were included through convenient and purposive sampling after taking informed consent. Sample size was calculated using the following formula.^18^


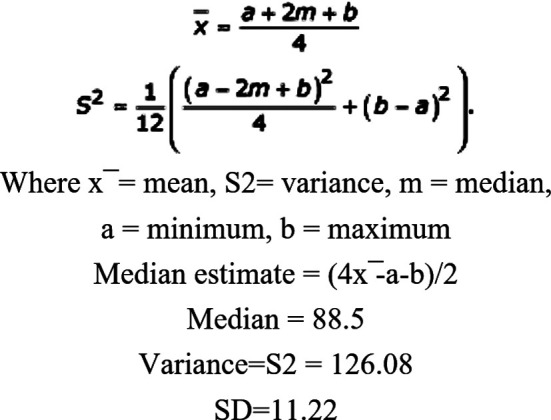


By using (x_1)® = 76.7, (x_2)® =88.4, SD=11.22, the estimated sample at 5% significance level and power of 90 was found to be 20 in each group.

After randomization by software (Random Allocation software version 1.0), all the volunteer fourth year medical students were divided into two groups. Both the groups were taught by the same teacher. A session on teaching concept map construction was taken with the control Group-A after the intervention and with experiment Group-B prior to intervention. Three teaching sessions of one hour each on the following selected topics (Jaundice in surgical patients, acute upper abdomen, Breast diseases) were scheduled for Experimental Group-B with concept map while the same topics were taught to the control Group-A using interactive methods such as questioning, clinical scenarios and power point presentation. Both Groups were taught by same teacher. Each group took a post class test using “One Best Type” challenge. Total scores of the two groups were compared for immediate effects on knowledge / short term knowledge retention.

### Statistical analysis:

Data analysis was carried out using SPSS version-21. Descriptive statistics were computed. Mean and standard deviation calculated for quantitative output response. Frequency percentage computed for qualitative output response. Post test scores of both groups were compared for any significant difference with unpaired student t test.

Statistical significance was taken at p<0.05.

### Inclusion criteria:

- All students of fourth year MBBS who volunteer to participate.

### Exclusion criteria:

- Those students who did not give consent to participate in the study or were unable to participate in any session / test

### Ethical Approval:

Study was approved by Clinical Research Committee and Ethical Review Committee Ziauddin Medical University reference code 0271215, Dated: 22-03-2016.

## RESULTS

A total of 60 students participated in our study, Including 14 (23.3%) males and 46 (76.7%) females. Out of 60 students, 31 (51.7) belonged to control Group-A while 29 (48.3) belonged to experimental Group-B. Our study reported a mean post test score of 19.5 (2.75 ± 0.49) for the control Group-A while 21.1 (3.49 ± 0.65) for experimental Group-B.

Independent Samples t-test was applied in two parts. The two parts provide different pieces of information: (A) Levene’s Test for Equality of Variances and (B) *t*-test for Equality of Means. The *p*-value of Levene’s test is reported to be 0.493 which is insignificant as the value is too large. In this case the null hypothesis of Levene’s test holds true and we can conclude that the variance in post-intervention test scores of students in Group-B is not significantly different than that of the control Group-A. This shows us that we should look at the “Equal variances assumed” row for the *t*-test results. Our study reported a negative *t* value of 2.003 with a significant p-value of 0.05 that means that the test scores of the control Group-A were lower than the scores of experimental Group-B.

## DISCUSSION

Our study reinforced CM as a meaningful learning strategy in clinical science with significantly higher scores in test. Our result were in line with many other studies similar to our study in science, nursing and basic medical education, giving our study a synergistic effect.^10^-^17^ However almost all of the comparative studies compared the concept mapping with traditional teaching strategy but in our study we compared both modern, active strategies based on constructivism. Additionally, for educators serving in medical schools, results of our study, and all other synergist studies suggest ideas on how to utilize concept mapping strategy in developing motivation, engagement and deep thinking in clinical setting, in enhancing students approach to solve problem based questions through critical thinking and in promoting concept mapping as a tool of collaborative learning. Studies have been performed to facilitate teachers and planners in constructing effective concept map structures that impart expert knowledge of the subject to the students, encourage peer teaching and allow learners to both view and reflect on their knowledge structures hence fostering metacognition and critical reflection. CMs can accompany students’ progress, enabling a graphical representation of knowledge.^19^

Choudhary et al. in their study concluded that concept maps are an effective tool for the formative assessment in Biology teaching at the secondary level. These are also helpful in eradicating misconceptions among students. Its use can be a valued addition in the process of teaching and learning science subjects. Concept maps must be used for teaching and learning.^20^

In a systemic review including thirty-nine studies from twenty-six journals, CMs were considered as a flexible tool facilitating knowledge integration in both learning and teaching method with positive perception from both students and tutors.^21^ Daley BJ et al reviewed 35 published studies from medical sciences and showed how concept maps can address four main purposes of medical education–promoting meaningful and integrated education, giving supplementary learning resources, aiding the teachers to deliver feedback to the students and assessing performance and improvement.^4^ Boman and Gul supported their findings by suggesting that a fifty-page word document can be summarized into a single page concept map, thereby, making it an effective and feasible method.^6^

A reasonable amount of enquiry has been done to see the effects of teaching with CM in nursing students, in a review from ten articles CM were found to be useful in active learning with the establishment of new knowledge and improvement in cognitive abilities.^10^ Experimental and quasi-experimental studies reviewed in a meta-analysis showed that students extending from Grade 4 to high school used CM to learn in areas such as psychology, science, statistics, and nursing learned by creating, modifying or inspecting node-link diagram. Out of fifty-five studies including 5,818 participants, 67 standardized mean difference effect sizes were retrieved, concluding that CM activities seems more effective for achieving knowledge transfer as well as retention compared to text passages reading, lectures and class discussions.^9^

In another study from Pakistan, conducted on 50 dental students randomly selected in control group trained by traditional methods and intervention group trained by concept maps post intervention results showed noteworthy difference between both the groups in one best type questions.^16^ CM is now quite often used as an educational tool in a diversity of classroom as well as professional settings. Researchers have used it for assessment as well. In a study, 21 pediatric resident completed CM training, represented a pre-instruction CM about ‘seizures’, completed a course on seizures and drew a post instruction CM on seizure. Using structural and relational methods two raters marked each map, a significant difference of scores was found in pre and post teaching CM.^22^

Studies are also conducted by incorporating concepts maps with problem based learning. In yet another study, medical undergraduates perceived concept mapping to be a helpful and effective teaching technique with results showing a noteworthy difference in Problem Based Questions score taught by CM in comparison to the topics learned by the traditional lectures.^15^ Similar results were found in this quasi-experimental study conducted on medical students, California Critical Thinking Skills Test score were high in intervention group (concept mapping) than control group (lecture-based).^17^

In another quasi-experimental study among 64 nursing students, crossover design was used. Group-A drew concept maps and Group-B was assessed by traditional assessment (quiz) for a duration of eight weeks. Both took a cumulative Test-I. Subsequently, both the groups used another technique for next eight weeks and took the cumulative Test-II.

The results from this study revealed that the mean marks for cumulative Test-I and Test-II were higher in that group which was involved in map construction and obtained higher mean scores for both cumulative tests as compared to the other group involved in quizzes only.^23^

Another study evaluating the role of CM technique in enhancing critical thinking among pediatric nursing trainees, the intervention group showed significantly greater critical thinking than in the control Group-After the program.^24^

### Limitation:

It includes single centre study with a limited sample size. We recommend further research in clinical sciences with a larger sample size to confirm our observations.

## CONCLUSION

Significant difference were observed in surgery exam scores in students taught by concept maps which adds to the results of current studies in supporting the promising role of CM.

### Authors’ Contributions:

***ZM:*** Conception & design, acquisition of data its analysis & interpretation. Preparing the manuscript

**MJ:** Substantial contributions to conception & design. Critical Review, preparation of manuscript.

**SMA and ZM:** Substantial contributions to analysis & interpretation of data. Review.

All authors have read the final version of manuscript and are accountable for the integrity of the study.
